# Deep-Fried Signaling: *tempura* Gets Notch Cooking

**DOI:** 10.1371/journal.pbio.1001778

**Published:** 2014-01-28

**Authors:** Caitlin Sedwick

**Affiliations:** Freelance science writer, San Diego, California, United States of America

As a multicellular animal's tissues mature throughout embryogenesis, its cells must communicate with each another to determine their appropriate placement and physiological role. In some cases, this communication takes place through long-range, secreted signals; in others, through direct cell-cell contact. One example of the latter case is a process called lateral inhibition, wherein a developing neuronal precursor cell (called a neuroblast) presents a signal to neighboring cells that prevents them from also adopting the neuroblast cell fate. To accomplish this, the neuroblast expresses on its surface a protein called Delta, which is a ligand for the receptor protein Notch. These receptors, expressed on neighboring cells, then signal to suppress the manufacture of neuroblast-specific genes in those cells. Thereafter, whenever the neuroblast divides, Notch signaling ensures that only one of the two daughter cells of that division retains the potential to become a neuron.

**Figure:**
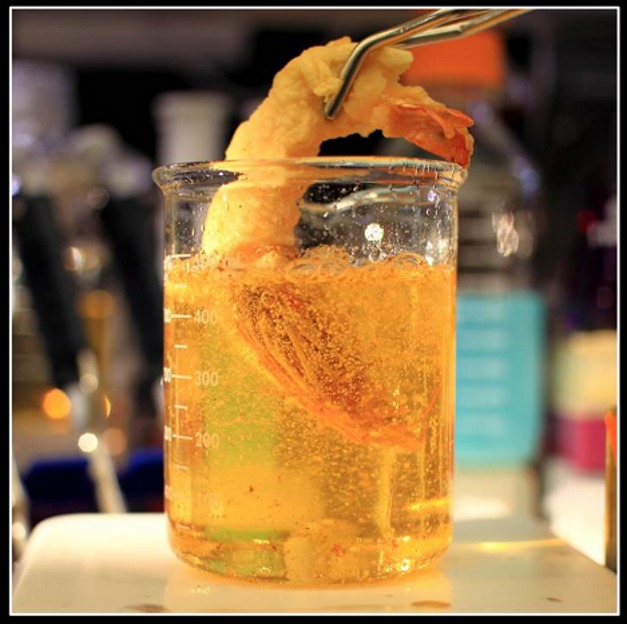
Frying Rabs with *tempura* for Notch signaling. In this issue, Charng and colleagues identify and characterize a gene that encodes a lipid transferase that is required for proper Notch signaling. They called this gene “*tempura*” after the Japanese deep fried dish; substrates, e.g., Rab-GTPases (depicted as a shrimp being submerged into laboratory equipment) have lipids attached by the Tempura protein. *Image credit: Kuchuan Chen, Karen Schulze, Wu-Lin Charng, Yuki Yamamoto, Shinya Yamamoto, Hector Sandoval, and Hugo Bellen.*

As may be expected, loss or alteration of Notch signaling has strong consequences for an embryo. But, not all the factors that affect the Notch pathway are known. So, in their paper published in this month's *PLoS Biology*, Wu-Lin Charng, Hugo Bellen, and colleagues set out to look for new proteins that influence Notch signaling.

To identify proteins involved in the Notch pathway, Charng and colleagues took advantage of a highly visible change caused by loss of Notch activity. If Notch signaling is blocked during fly development, it leads to a failure of lateral inhibition and cell fate specification during external sensory organ (ESO) development. As a result, cells that would normally have formed sensory hairs and associated structures on the back of the animal's thorax instead become neurons. Flies with this “bald” phenotype are easily distinguished from normal flies. The authors used this characteristic to select animals with Notch pathway defects amongst the progeny of flies that had been exposed to a chemical mutagen.

Using this screening process, Charng and colleagues identified a gene whose loss causes a strong balding phenotype. Genetic mapping showed that this gene isn't one of the proteins already known to affect Notch signaling. Sequence analysis showed that the protein likely has the ability to add lipid moieties to other proteins, leading the researchers to dub this new gene “*tempura*” (*temp* for short), after the deep-fried Japanese dish. The group then set out to characterize it.

The first thing that caught the authors' attention about *tempura* was that the phenotype of *temp* mutant animals resembles that of animals with mutations in another gene, called *scabrous*. Produced by neuroblasts in the developing ESO, Scabrous is a secreted protein that enhances Notch signaling in surrounding cells. Charng and coworkers noticed that *temp* mutant ESOs show a strongly elevated expression of Scabrous, therefore *temp* flies do not get their balding phenotype because of impaired Scabrous expression. Instead, the group found that the cells in *temp* mutant ESO cells fail to secrete the Scabrous they make; the protein accumulates inside the cells' Golgi bodies. This, in turn, suggested that *temp* could be involved in mediating proper trafficking of intracellular vesicles needed for Scabrous secretion. Consistent with the idea that mutations in *temp* are involved in vesicular trafficking, the authors also found that *temp* mutant ESO cells have impaired surface expression of the Notch ligand, Delta. Furthermore, Delta and Scabrous were frequently found trapped in the same intracellular structures.

For clues about how *temp* affects vesicular trafficking, Charng and colleagues took a closer look at the gene's sequence. As already mentioned, Temp's predicted amino acid sequence suggested that the *temp* gene encodes a protein with the ability to add lipid modifications to other proteins. In fact, the authors found that Temp protein can act as an alternative subunit in a protein complex that is known to add lipids specifically to proteins of the Rab family. As part of this complex, Temp can mediate the addition of lipids to two particular Rabs, Rab1 and Rab11. When Temp is unavailable, its Rab targets don't get their proper lipid modifications—causing them both to become mislocalized within the cell. Rabs are well known to be coordinators of vesicular trafficking, so this explains why *temp* mutant cells don't properly traffic Scabrous and Delta: Scabrous and Delta rely on Rabs to get where they need to go, but the Rabs themselves are sidelined in the absence of Temp.

While Rab11 was already known to be involved in trafficking of Delta-containing intracellular vesicles, Rab1 had never before been connected to the Notch pathway. This new connection could have interesting implications for our understanding of Notch signaling in the many tissues and cells where Notch helps determine cell fate. Furthermore, the authors' identification of *temp*'s role in Rab lipid modification has uncovered some unanticipated complexity in the regulation of Rab activity and of vesicular trafficking in general.

**Charng W-L, Yamamoto S, Jaiswal M, Bayat V, Xiong B, et al. (2014)** ***Drosophila*** **Tempura, a Novel Protein Prenyltransferase α Subunit, Regulates Notch Signaling Via Rab1 and Rab11.** doi:10.1371/journal.pbio.1001777

